# Simple Tyrosine Derivatives Act as Low Molecular Weight Organogelators

**DOI:** 10.1038/s41598-019-41142-z

**Published:** 2019-03-20

**Authors:** Güzide Aykent, Cansu Zeytun, Antoine Marion, Salih Özçubukçu

**Affiliations:** Middle East Technical University, Department of Chemistry, 06800 Ankara, Turkey

**Keywords:** Computational methods, Molecular self-assembly, Gels and hydrogels

## Abstract

The gelation of L-Tyr(*t*Bu)-OH in tetrahydrofuran (THF) was discovered serendipitously. It was noted that this tremendously low molecular weight (LMW) compound has the ability to gel a wide variety of organic solvents (e.g., *N,N*-Dimetylformamide (DMF), THF, butanol, toluene), even in very low concentrations (i.e., 0.1 wt/v% in DMF). Addition of bases such as NaOH and piperidine enhanced the gel property. By changing the side-chain protecting group to *tert*-butyldimethylsilyl (TBDMS), a fluoride ion-responsive organogel was also acquired. This new organogelator responded fluoride ion concentration as low as 0.2 ppm. Characterization of microstructures and gel behaviours were studied by powder X-Ray diffraction spectroscopy (XRD), transmission electron microscopy (TEM), rheological measurements and molecular dynamics (MD) simulations. Experimental observations and theoretical simulations consistently show a fibre-like structure of the gel, in which the organogelator molecules are held together via a dense network of hydrogen bonds, and via van der Waals interactions between hydrophobic groups.

## Introduction

Low molecular weight (LMW) organogels have attracted significant research attention over the past two decades due to their numerous potential applications, including drug delivery, oil-spill recovery, smart electronics, and stimuli-responsive materials^1–5^. Characteristics such as the intermolecular hydrogen bonding, π-π stacking of aromatic units, hydrophobic effect, and van der Waals forces lead to the self-assembly of low molecular weight organogelators (LMWO) in fibrous structures, tapes, sheets, etc^1,6^. The entanglement of these secondary structures eventually results in the immobilization of the solvent, which can be referred to as a gel^7^. Amino acids are versatile compounds for self-assembled structures and, therefore, have found various applications as LMWO^8–14^. However, amino acids usually suffer a lack of wide range solvent applicability or require several steps to synthesize. There are also many examples of multi-component LMW gels in the literature^15^. In some cases, two components are necessary to form a gel, whereas in other cases two different components, each having gelation ability on its own, are mixed in order to obtain a gel that results from the assembly of the two components in an alternating fashion. Additionally, a non-gelling additive can be used to increase the lifetime stability of a gel and/or to enhance its mechanical properties.

The design of new fluoride-sensitive molecules is currently the focus of research efforts on the part of many groups, notably due to the implications of such molecules for dental care and for the treatment of osteoporosis^16–18^. However, overexposure to fluoride ions can cause kidney problems and fluorosis. The World Health Organisation has declared the appropriate amount of fluoride ions in drinking water to be 0.5–1.0 mg/L^19^. In the literature, stimuli-responsive organogels have been used to selectively detect fluoride ions by forming charge-transfer complexes^20–23^. Further, many chemosensor systems have been investigated with regards to their ability to sense fluoride ions using the fluorescence and colorimetric properties of materials^16,17^. Some of these studies depend on the cleavage of the silicon-oxygen bond of silyl ethers to promote fluoride ions^24–28^. In the present study, we demonstrated the organogelator properties of L-Tyr(*t*Bu)-OH, a tremendously low molecular weight organogelators. This organogelator has a molecular weight of only 237 amu, as well as an outstanding gelation ability in relation to a wide range of organic solvents down to a concentration of 0.1 wt/v%. Then, with regards to potential chemosensor applications, we synthesized L-Tyr(TBDMS)-OH and investigated its gelation properties under various conditions. L-Tyr(TBDMS)-OH is sensitive to the presence of fluoride, since the latter can trigger the cleavage of the Si-O bond to form L-Tyr-OH.

## Results and Discussion

### Spontaneous Gelation of L-Tyr(*t*Bu)-OH (2)

**Figure 1:**
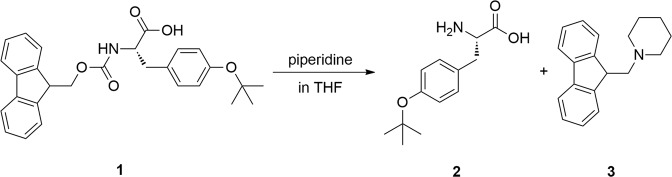
Synthesis of L-Tyr(*t*Bu)-OH.

L-Tyr(*t*Bu)-OH is an inexpensive commercially available compound and is also synthetically straightforward to produce quantitatively in one step from Fmoc-L-Tyr(*t*Bu)-OH^29^. Gelation occurs spontaneously during the following reaction (Fig. 1).

The cleavage of the fluorenylmethyloxycarbonyl (Fmoc) group with piperidine in THF resulted in gelation within 15 minutes during the reaction. After the removal of the by-product, gelation was successfully achieved under the same conditions using pure L-Tyr(*t*Bu)-OH which indicates that the by-product does not play a role in the gelation process. This reaction was initially performed aiming to synthesize unprotected tyrosine to be used in another study and eventually led us to the serendipitous discovery of this new organogelator, which triggered further investigations as reported hereafter.

Same gelation properties were observed for the other enantiomer, D-Tyr(*t*Bu)-OH, as expected. However, no gel formation was observed in the case of racemic Tyr(*t*Bu)-OH at minimum gelation concentration. In addition, we observed that the *tert*-butyl moiety plays a significant role in the gelation process, since gel formation does not occur for either L-Tyr-OH, L-Phe-OH and L-Tyr(Me)-OH.

### Additive and solvent screening for gelation

In order to investigate the gelation properties of L-Tyr(*t*Bu)-OH, different combinations of additives and solvents were considered using the vial inversion method (Tables 1 and 2). Table 1 summarizes the effects of the different additives (bases and alcohols) on gelation in THF.

**Table 1:** Gelation results of L-Tyr(tBu)-OH with different additives in THF.

Additives	Conc. (wt/v%)	Appearances
—	1.1^a^	Gel
Piperidine	0.45^a^	Gel
Diisopropylamine	1	Gel
Diethylamine	1	Gel
DBU	1	Solution
Imidazole	2	Gel
2-ethylhexylamine	1	Solution
Triethylamine	1	Suspension
NaOH(aq)	0.25^a^	Gel
Methanol	2	Suspension
Ethanol	2^a^	Gel
Isopropanol	2^a^	Gel
*n*-Butanol	1.5^a^	Gel
2-ethylhexanol	0.75^a^	Gel

^a^Minimum gelation concentration.

**Table 2:** Gelation results of L-Tyr(*t*Bu)-OH in different solvents with and without non-gelling agent. The values are given in wt/v%.

Solvent Additive	DMF	2-EtHex	Toluene	MTBE	1, 2-DME	ACN	Hexane	1, 2-DCE	IPA	*n*-BuOH	Sunflower oil	Cellulose Thinner	Diesel
none	0.1	0.2^a^	sol (1.0)	0.7^a^	0.3^a^	0.3^a^	sol (0.75)	sol (1.0)	0.5^a^	0.5^a^	ppt (1.0)	1.0	sol (2.0)
piperidine	*nt*	0.2^a^	0.3^a^	0.45^a^	0.2^a^	sol (0.2)	0.75	0.2^a^	sol (0.4)	sol (0.4)	1.0	sol (1.0)	2.0
NaOH	*nt*	*nt*	ppt (1.0)	ppt (1.0)	0.4^a^	ppt (0.2)	ppt (1.0)	ppt (1.0)	*nt*	sol (0.4)	ppt (1.0)	ppt (1.0)	ppt (2.0)

DMF: *N,N*-Dimethylformamide; 2-EtHex: 2-Ethylhexanol; MTBE: *tert*-Butylmethylether; 1,2-DME: 1,2-Dimethoxylethane; ACN: Acetonitrile; 1,2-DCE: 1,2-Dichloroethane; IPA: isopropylalcohol; *n*-BuOH: *n*-Butylalcohol; *nt*: not tested; sol: solution; ppt: precipitate; ^a^Minimum gelation concentration.

Gels were prepared in 1.0 mL of THF. After dissolution of L-Tyr(*t*Bu)-OH in solvent with the help of ultrasonic bath at 40 °C, 10.0 μL of additive was added. The solutions were placed again into ultrasonic bath for 4–10 minutes. The formation of the gels was determined using inversion test.

L-Tyr(*t*Bu)-OH forms a gel in THF with a minimum concentration of 1.1 wt/v% without the use of any additive. Surprisingly, the addition of piperidine lowers the minimum gel concentration to 0.45 wt/v%. Gelation also occurs in the presence of other bases, such as diisopropylamine, diethylamine and imidazole, albeit with weaker gel appearances. However, when triethylamine and DBU is used as additive, a suspension and a clear solution is formed, respectively. This result suggests that the additive should also have donor-acceptor hydrogen bond ability to trigger gel formation. To further test this hypothesis, short-alkyl-chain-containing alcohols were also considered but were found to play no favourable role in the gelation process as an additive. On the other hand, 2-ethylhexanol does show significant effect on gelation, which indicates that van der Waals interactions between additive and organogelator also decrease the minimum gelation concentration. Beside these organic bases and alcohols, the addition of 1 eq. of NaOH resulted in a minimum gelation concentration as low as 0.25 wt/v% in THF. These results show that additives are not necessarily involved in the hydrogen bond network, but that the amine-type additives are also acting as a base, which deprotonates the carboxylic acid, allowing the resulting carboxylate group to better participate in the hydrogen bond network. This is also consistent with the higher minimum gelation concentrations observed when short-alkyl-chain-containing alcohols were used as additives, since alcohols are weakly acidic.

The gelation of L-Tyr(*t*Bu)-OH was further tested in a wide variety of solvents with or without the addition of the non-gelling agent, piperidine or NaOH. As shown in Table 2, L-Tyr(*t*Bu)-OH forms a gel with a wide variety of organic solvents at remarkably low concentrations.

Among the tested conditions, DMF appears as the best solvent for gelation, with an ability to gel at 0.1 wt/v% without any additive. Further, 2-ethylhexanol, when used as a solvent, shows promising gelation results with a concentration as low as 0.2 wt/v%. The addition of a non-gelling agent to the latter gel merely changes the appearance of the gel to transparent and does not have a significant impact on the minimum gelation concentration. Similarly, toluene, hexane, and 1,2-dichloroethane also appear to be good solvents for gelation only with the addition of piperidine. Presence of piperidine decreases the gelation concentration for the solvents *tert*-butylmethylether and 1,2-dimethoxyethane, of which they formed solution at the defined concentrations without additives. Surprisingly, cellulose thinner, isopropylalcohol, and *n*-butylalcohol, when used as solvents, result in a clear gel without piperidine, whereas the addition of piperidine forms solutions. The gelation of sunflower oil indicates a promising potential application of L-Tyr(*t*Bu)-OH in the field of drug delivery^30–32^. Similarly, the gelation of diesel by L-Tyr(*t*Bu)-OH was observed, indicating a possible function in oil-spill recovery^3,10^.

### Gelation and Fluoride Ion Response of L-Tyr(TBDMS)-OH

**Figure 2:**
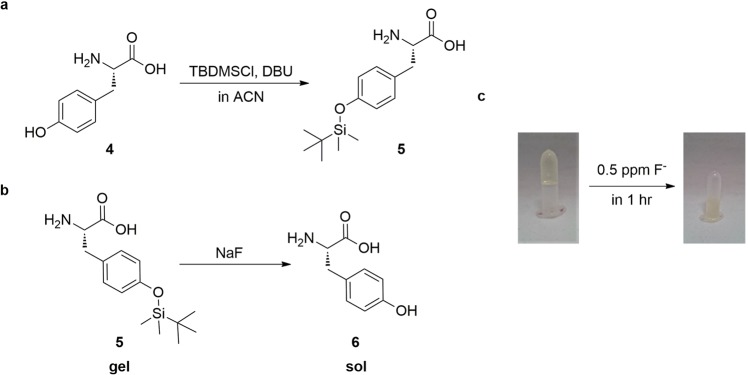
(**a**) Synthesis of L-Tyr(TBDMS)-OH (**b**) Si-O bond cleavage of L-Tyr(TBDMS)-OH in the presence of fluoride ion. (**c**) Image of complete gel to solution transition of 2 wt/v% of L-Tyr(TBDMS)-OH in 2-ethylhexanol as solvent and piperidine as additive after 1 hour upon addition of 0.5 ppm NaF(aq.)

We synthesized L-Tyr(TBDMS)-OH (Fig. 2a) and investigated its gelation properties under various conditions. The gelation of this derivative was achieved in both THF and 2-ethylhexanol, with a minimum concentration of 1 wt/v% in both solvents. L-Tyr(TBDMS)-OH is sensitive to the presence of fluoride, since the latter can trigger the cleavage of the Si-O bond to form L-Tyr-OH, as depicted in Fig. 2b. The addition of sodium fluoride to L-Tyr(TBDMS)-OH gels in 2-ethylhexanol in concentrations of 0.2, 0.3 and 0.5 ppm resulted in a complete gel to solution transition within 44 h, 18 h, and 1 h, respectively (Fig. 2c). Fluoride ion cleave the TBDMS moiety to yield L-Tyr-OH, which, as previously discussed, does not show gelation properties in 2-ethylhexanol, explaining the gel to solution transition observed after a certain period of time. We therefore suggest L-Tyr(TBDMS)-OH as a potentially promising gelator that can be used for the detection of fluoride ions.

### Characterization of Microstructures and Gel Behaviours

**Figure 3:**
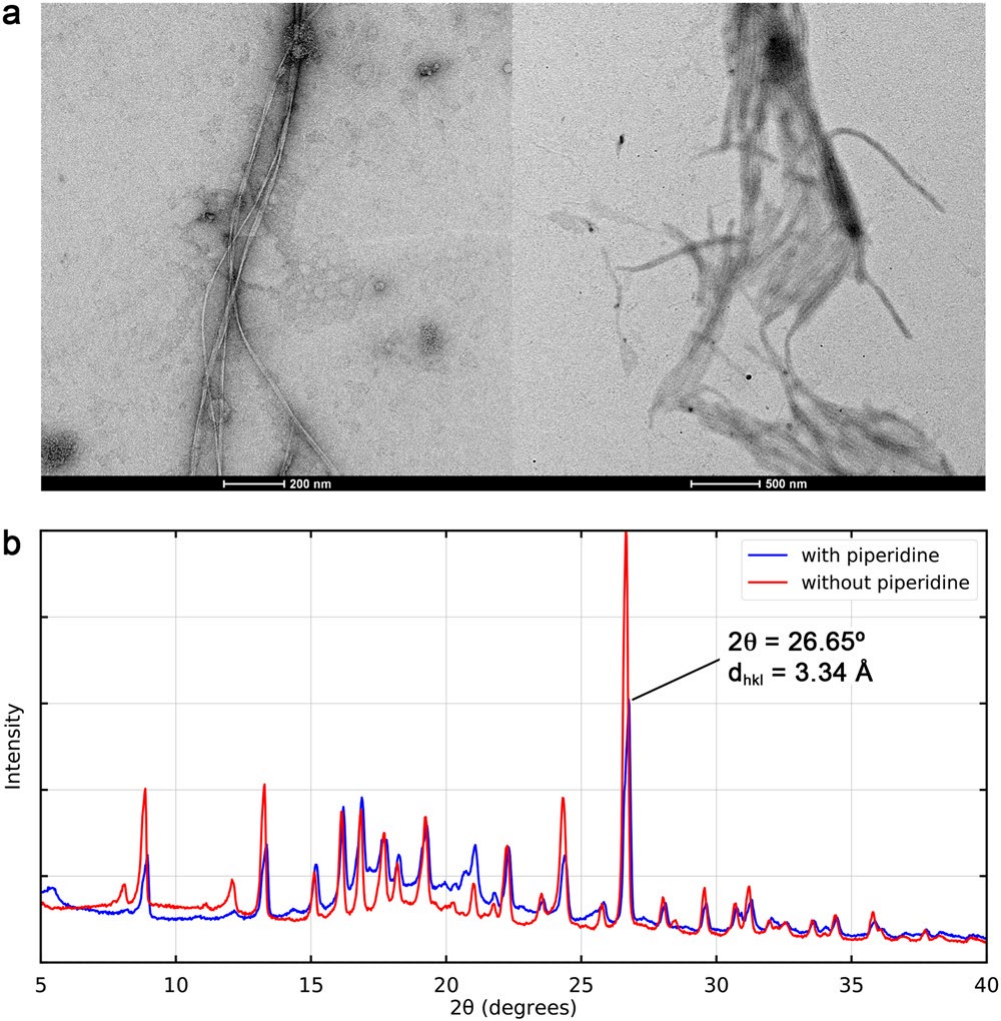
(**a**) TEM images of L-Tyr(*t*Bu)-OH in THF/piperidine diluted with miliQ water. (Scale bar: 200 nm and 500 nm). (**b**) XRD pattern of xerogels with and without piperidine.

The characterization of the microstructure of the L-Tyr(*t*Bu)-OH gel as well as the molecular packing at an atomic scale was performed using transmission electron microscope (TEM) imaging (Fig. 3a), X-ray powder diffraction (XRD) measurements (Fig. 3b), and molecular dynamics (MD) simulations.

In Fig. 3a, the TEM images of the L-Tyr(*t*Bu)-OH in THF with the addition of piperidine show the formation of nanofibres with an approximate width of 40 nm and a length of several micrometres. The XRD patterns reported in Fig. 3b for the samples prepared with and without piperidine additive indicate that piperidine does not participate in the molecular packing, since the two patterns are nearly identical.

MD simulation of L-Tyr(*t*Bu)-OH in THF resulted in the spontaneous formation of aggregates stabilized by strong hydrogen bond interactions between the carboxylate and ammonium groups of the gelator (Fig. S7a). A similar simulation including piperidine molecules also resulted in a spontaneous self-assembly, though with a significantly different interaction motif (Fig. S7b).

**Figure 4:**
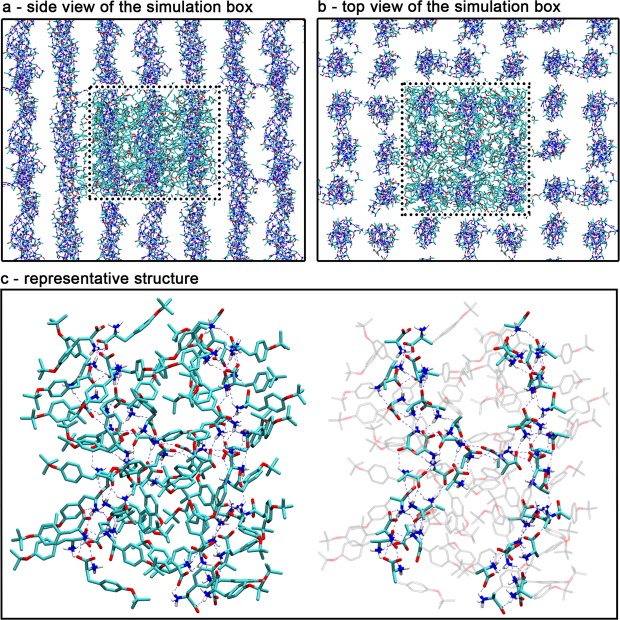
Representation of the fibre-like structure as obtained after one microsecond MD simulation of pure L-Tyr(*t*Bu)-OH. (**a**,**b**) Respectively show a side and top view of the simulation box with its periodic images. The dotted lines delimit the main unit cell in which backbone and side chains carbon atoms of each molecule are depicted in cyan. Only the backbone atoms, forming the core of each fibre are shown for the replica to highlight the linear structure of the different assemblies. A representative structure of two interacting fibres is shown in (**c**), with two representations to show the whole molecules on the left-hand side and to highlight the interactions in the core of the fibres only on the right-hand side. Hydrogen bonds are represented with dashed blue lines and show the network by which the individual molecules interact to form fibres and punctually bridge them together.

As such a different molecular packing would result in a different XRD pattern, we therefore definitely rule out the structural role of piperidine in the formation of the gel. The corresponding results are further discussed in the Supporting Information. The subsequent microsecond-long MD simulation of pure L-Tyr(*t*Bu)-OH highlights the formation of long networks of interacting molecules, which form a series of parallel packed fibre-like structures (Fig. 4), in agreement with the TEM imaging.

As highlighted in Fig. 4c and further detailed in Fig. S7, each fibre is composed of a hydrophilic core with a strong and compact network of hydrogen bonds, while the hydrophobic side chains point outward and ensure interactions between fibres. We also noted the sporadic occurrence of hydrogen bonds branching between the fibres, which are likely to participate in the overall stability of the molecular assembly. The analysis of radial distribution functions (RDF; Fig. S8) for specific pairs of atoms along the dynamics revealed a series of well-defined peaks centred at distances consistent with the diffractions seen in the XRD pattern. Our simulations showed no evidence of stable π−π stacking interaction between aromatic rings of L-Tyr(*t*Bu)-OH, which is most likely due to the large steric hindrance by *t*Bu groups. Instead, the sharp and intense peak at 2θ = 26.65° (dhkl = 3.34 Å) in the XRD pattern can be attributed to the tight interaction observed between carboxylate and ammonium groups along the simulation, with a carbon-nitrogen RDF strongly peaking at a distance of 3.33 Å. Additional discussions, simulations details, and parameter files are also available in the Supporting Information.

The gelation process is triggered by driving forces similar to those at play in the formation of reverse micelles. The hydrophobic character of the solvent enhances the tendency of the polar part of L-Tyr(*t*Bu)-OH molecules to interact with each other, in an isolated core with hydrophobic *t*Bu groups pointing towards the solvent. Unlike micelles, however, the small size of the hydrophilic amino acid backbone together with the highly directional and dipolar character of the interacting chemical groups favours the formation of a linear network rather than spherically shaped vesicles. As predicted by MD simulations (Figs 4 and S8) this linear network can branch through the interconnection of fibres via hydrogen bonds and van der Waals interaction. This last observation leads us to postulate that the gelation results from the formation of a three-dimensional grid of interconnected fibre-like structures in solution.

In summary, L-Tyr(*t*Bu)-OH and L-Tyr(TBDMS)-OH are forming gels due to a dense network of hydrogen bonds between ammonium and carboxylate groups. A number of amino acid-derived organogelators have been reported in the literature, presenting substitution either on the amine or on the carboxyl group. To the best our knowledge, however, there was thus far no report of a natural amino acid-derived organogelator bearing free -NH_2_ and –COOH groups, which we showed here to be the main factor affecting the three-dimensional structure of the gel. Also, *tert*-butyl moiety appears to play a crucial role in the gelation process by preventing the π−π interactions due to its steric hindrance. This is further showed by the incapability of L-Tyr(OMe)-OH to form a gel. The methyl group seems to be too small to prevent the π−π interactions, leading to precipitation instead of gelation in THF.

**Figure 5:**
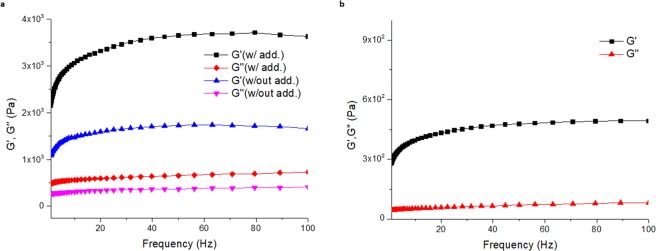
Viscoelastic behaviour of (**a**) 1 wt/v% L-Tyr(tBu)-OH in 2-ethylhexanol and 2-ethylhexanol/piperidine; (**b**) 1 wt/v% L-Tyr(TBDMS)-OH in 2-ethylhexanol/piperidine.

Rheological measurements were conducted to investigate the gel behaviours of both compounds, L-Tyr(*t*Bu)-OH and L-Tyr(TBDMS)-OH (Fig. 5). A large difference between dynamic storage modulus G″ and loss modulus G′ at all frequencies indicates that the organogels in question show elastic character as a soft matter dominantly. To prove the favourable effect of non-gelling additive on the gel, piperidine added gel was also investigated (Fig. 5a). Although we showed that the piperidine additives did not participate in the molecular structure of the gel, the rheological results clearly showed that such non-gelling additives enhanced the gel properties. The greater difference between G″ and G′ for the piperidine containing organogel compared to additive free organogel leads us to conclude that the presence of non-gelling additive enhanced the gel properties of these soft material.

## Conclusion

The present work reports the serendipitous discovery of a new LMW organogelator, L-Tyr(*t*Bu)-OH, which is an inexpensive compound commercially available from many common vendors and which can be synthesized in a straightforward manner from Fmoc-L-Tyr(*t*Bu)-OH in one step. To the best of our knowledge, L-Tyr(*t*Bu)-OH currently represents one of the lightest LMW organogelator to have been reported in the literature and the only natural amino acid-derived gelator having free –NH_2_ and –COOH groups. Other amino acid-derived organogelators known in the literature form gel in limited types of solvents or require synthesis involving several steps. Our analysis showed that the molecule has the ability to form a gel in a wide range of organic solvents (polar, protic, apolar etc.), with a minimum gelation concentration as low as 0.1 wt/v% in *N,N*-dimethylformamide. Its gelation in sunflower oil and diesel indicates promising potential for applications in the fields of drug delivery and oil-spill recovery, respectively. We also found that the addition of a non-gelling additive, such as piperidine or NaOH, lowers the minimum gelation concentration and increases the mechanical properties of the gel without any disruption of the molecular arrangement. Additionally, we synthesized another new organogelator, L-Tyr(TBDMS)-OH, in a single step from L-Tyr-OH. The *O*-silylation of tyrosine is much simpler than alkylation, because *O*-alkylation of L-Tyr-OH requires extra protection of COOH and NH_2_ groups which makes the synthesis quite long. While slightly heavier than L-Tyr(*t*Bu)-OH, this new low molecular weight organogelator demonstrates great potential of being used as a chemosensor for the detection of fluoride ions in drinking water in ppm quantities.

## Methods

### Materials

All reagents are commercially available and used without any further purification. Fmoc-L-Tyr(*t*Bu)-OH, Fmoc-D-Tyr(*t*Bu)-OH and L-Tyr-OH were purchased from Chem-Impex International Inc.; *tert*-Butyldimethylchlorosilane from TCI Chemicals; DMF, 1,2-DME, imidazole, methanol and ethanol from Sigma Aldrich; THF, Toluene, MTBE, 1,2-DCE, *n*-BuOH, diisopropylamine and DBU from Merck; ACN, triethylamine from Carlo Erba Reagents; Piperidine and 2-ethylhexylamine from Acros Organics; hexane and isopropylamine from Lab Scan; diethylamine from Riedel-de Haen; 2-ethylhexanol from Veskim.

### Synthesis of L-Tyr(TBDMS)-OH (2)

*L-Tyr(TBDMS)-OH* was synthesised based on the literature synthesis of L-DOPA(TBDMS)_2_-OH with slight modifications^33^. TBDMS-Cl (1.20 g, 8.28 mmol) dissolved in anhydrous MeCN (12.5 mL) was added on L-Tyr-OH (0.100 g, 5.05 mmol). The mixture was cooled on an ice-water bath for 10 minutes. Then DBU (1.24 mL, 8.28 mmol) was added dropwise to the reaction mixture over 10 minutes. The reaction was stirred in ice bath for 4 hours and after that an additional 20 hours at room temperature. Then, the solvent was removed under vacuum. When methanol was added, undesired precipitate was formed. After filtration, solvent was removed under vacuum. The crude product was washed with water then, with ethyl acetate to obtain pure product. The proton and carbon NMR is shown in Figs S4 and S5, respectively.

^1^H NMR (400 MHz, CD_3_OD) δ 7.00 (2 H, d, *J* = 8.5 Hz), 6.63 (2 H, dd, *J* = 6.6, 1.9 Hz), 3.55 (1 H, dd, *J* = 8.7, 4.3 Hz), 3.05 (1 H, dd, *J* = 14.6, 4.2 Hz), 2.75 (1 H, dd, *J* = 14.6, 8.7 Hz), 0.80 (9 H, s), 0.00 (6 H, s); ^13^C NMR (100 MHz, CD_3_OD) δ 174.0 (1 C), 156.0 (1C), 131.6 (2C), 130.0 (1C), 121.5 (2C), 57.6 (1C), 37.5 (1C), 26.2 (3C), 19.0 (1C), −4.31 (2C). HRMS: calculated for [M-H]^+^ C_15_H_26_NO_3_Si, 296.1682; found 296.1696. Purity measured by HPLC (Fig. S6): 98.3%.

### General Gelation Procedure

In order to prepare 1% (w/v) gel; 10 mg L-Tyr-(*t*Bu)-OH was weighed and placed into a vial. 1.0 mL solvent was added. The vial was placed into ultrasonic bath for 4 minutes. Then 10.0 μL additive was added for the additive containing gels. Eppendorf tube was again placed into ultrasonic bath for 4–10 minutes. The temperature of ultrasonic bath is around 40 °C. Gel formation occurs spontaneously or up to 1 hour depending on the solvent used. Gel formations were proven by using inversion test.

### Fluoride Ion Response of gels of L-Tyr(TBDMS)-OH

After forming gel in 2-ethylhexanol as 1 wt/v%, 10 μL sodium fluoride solution was dropped on gel from the stock solution (5 mg NaF in 1 mL water) to analyze the effect of 0.5 ppm NaF. Then, it was allowed to stay without stirring. Complete gel to solution transition was observed with naked eye and inversion test.

### Transmission Electron Microscopy Imaging

FEI Tecnai G2 Spirit BioTwin CTEM microscope was used to image the fibrilar formations after self-assembly. 1 wt/v% gel of L-Tyr(*t*Bu)-OH and Tyr(TBDMS)-OH were prepared freshly in THF/piperidine. After diluting 50-fold with water, it was applied on Cu grid. Excess solution was removed after 2 minutes and grid was stained with 2% uranyl acetate solution.

### Rheological Measurements

1 wt/v% gels of L-Tyr(*t*Bu)-OH were prepared in 2-ethylhexanol with and without piperidine addition freshly for the rheological measurements. Similarly, 1 wt/v% gel of L-Tyr(TBDMS) was also prepared to investigate its rheological behavior. Physica MCR 301, Anton Paar was used. At first, the linear viscoelastic regimes of deformations of the organogels were determined by a strain-sweep experiment. With a limit of linearity of G′, the strain values that used for the strain-controlled analyses were assigned. Then, the visco-elastic character of the samples was examined by dynamic storage modulus G″ and loss modulus G′. Frequency sweep was scanned from 0.1 to 100 Hz by using a constant target strain determined before.

### Powder X-Ray Diffraction Measurements

XRD measurements were conducted using X’Pert³ MRD with Cu Kα X-ray radiation (λ = 1.540598 Å). Gels of L-Tyr(*t*Bu)-OH in THF and THF/piperidine were prepared as 3 wt/v% and allowed to dry in air for overnight and then vacuum was applied to obtain xerogels.

## Supplementary information

Supplementary Information

Supplementary Information

Supplementary Information

Supplementary Information

Supplementary Information

Supplementary Information

## Data Availability

The datasets generated during and/or analysed during the current study are available from the corresponding author on reasonable request.
