# Publisher Correction: Simple Tyrosine Derivatives Act as Low Molecular Weight Organogelators

**DOI:** 10.1038/s41598-019-43070-4

**Published:** 2019-05-03

**Authors:** Güzide Aykent, Cansu Zeytun, Antoine Marion, Salih Özçubukçu

**Affiliations:** Middle East Technical University, Department of Chemistry, 06800 Ankara, Turkey

Correction to: *Scientific Reports* 10.1038/s41598-019-41142-z, published online 20 March 2019

The incorrect Supplementary Information file was originally published with this Article. The correct Supplementary Information file now accompanies the Article.

