# Supplementary material for: Simple Tyrosine Derivatives Act as Low Molecular Weight Organogelators
Source: Sci Rep. 2019 Mar 20;9:4893. doi: 10.1038/s41598-019-41142-z (PMC6426947; doi:10.1038/s41598-019-41142-z)
Supplement: Supplementary file 1 — Supplementary Information [file 41598_2019_41142_MOESM1_ESM.pdf]

Simple Tyrosine Derivatives Act as Low Molecular Weight Organogelators

Güzide Aykent[1], Cansu Zeytun[1], Antoine Marion[1], Salih Özçubukçu[1*]

[1]Middle East Technical University, Department of Chemistry, 06800, Ankara, Turkey.

Electronic Supplementary Information (14 pages)

1. *Materials* 2
2. *Synthesis of L-Tyr(tBu)-OH* 2
3. *^{1}H and ^{13}C NMR Spectra of L-Tyr(tBu)-OH* 3
4. *HPLC Analysis of L-Tyr(tBu)-OH* 5
5. *^{1}H and ^{13}C NMR Spectra of L-Tyr(TBDMS)-OH* 6
6. *HPLC Analysis of L-Tyr(TBDMS)-OH* 8
7. *Modelling details and discussions* 9

References 15

1. *Materials*

All reagents are commercially available and used without any further purification. Fmoc-L-Tyr(*t*Bu)-OH, Fmoc-D-Tyr(*t*Bu)-OH and L-Tyr-OH were purchased from Chem-Impex International Inc.; *tert*-Butyldimethylchlorosilane from TCI Chemicals; DMF, 1,2-DME, imidazole, methanol and ethanol from Sigma Aldrich; THF, Toluene, MTBE, 1,2-DCE, *n*-BuOH, diisopropylamine and DBU from Merck; ACN, triethylamine from Carlo Erba Reagents; Piperidine and 2-ethylhexylamine from Acros Organics; hexane and isopropylamine from Lab Scan; diethylamine from Riedel-de Haen; 2-ethylhexanol from Veskim.

2. *Synthesis of L-Tyr(tBu)-OH*

L-Tyr(tBu)-OH was synthesised based on the literature procedure[1] with some modifications. To a solution of 1.0 g (2.2 mmol) of Fmoc-L-Tyr(*t*Bu)-OH in 16 mL of THF, 1.0 mL (11 mmol) of piperidine was added. After stirring for 3 hours at room temperature, solvent was removed under vacuum. Remaining white solid was washed with 200 mL of hexane in 10 portions and allowed to dry open to air to obtain white solid. The proton and carbon NMR is shown in Figure S1 and S2, respectively. ^{1}H NMR (400 MHz, CD_3OD) δ 7.36 – 7.12 (2H, m), 7.12 – 6.87 (2H, m), 3.77 (1H, dd, J = 8.8, 4.3 Hz), 3.29 (1H, dd, J = 8.8, 4.3 Hz), 2.99 (dd, J = 14.6, 8.8 Hz), 1.36 (9H, s); ^{13}C NMR (100 MHz, CD_3OD δ 173.9 (1C), 155.9 (1C), 132.3 (1C), 131.1 (2C), 125.6 (2C), 79.6 (1C), 57.6 (1C), 37.6 (1C), 29.2 (9C). Purity measured by HPLC (Figure S3): 98.9 %.

3. ^{1}H and ^{13}C NMR Spectra of L-Tyr(tBu)-OH

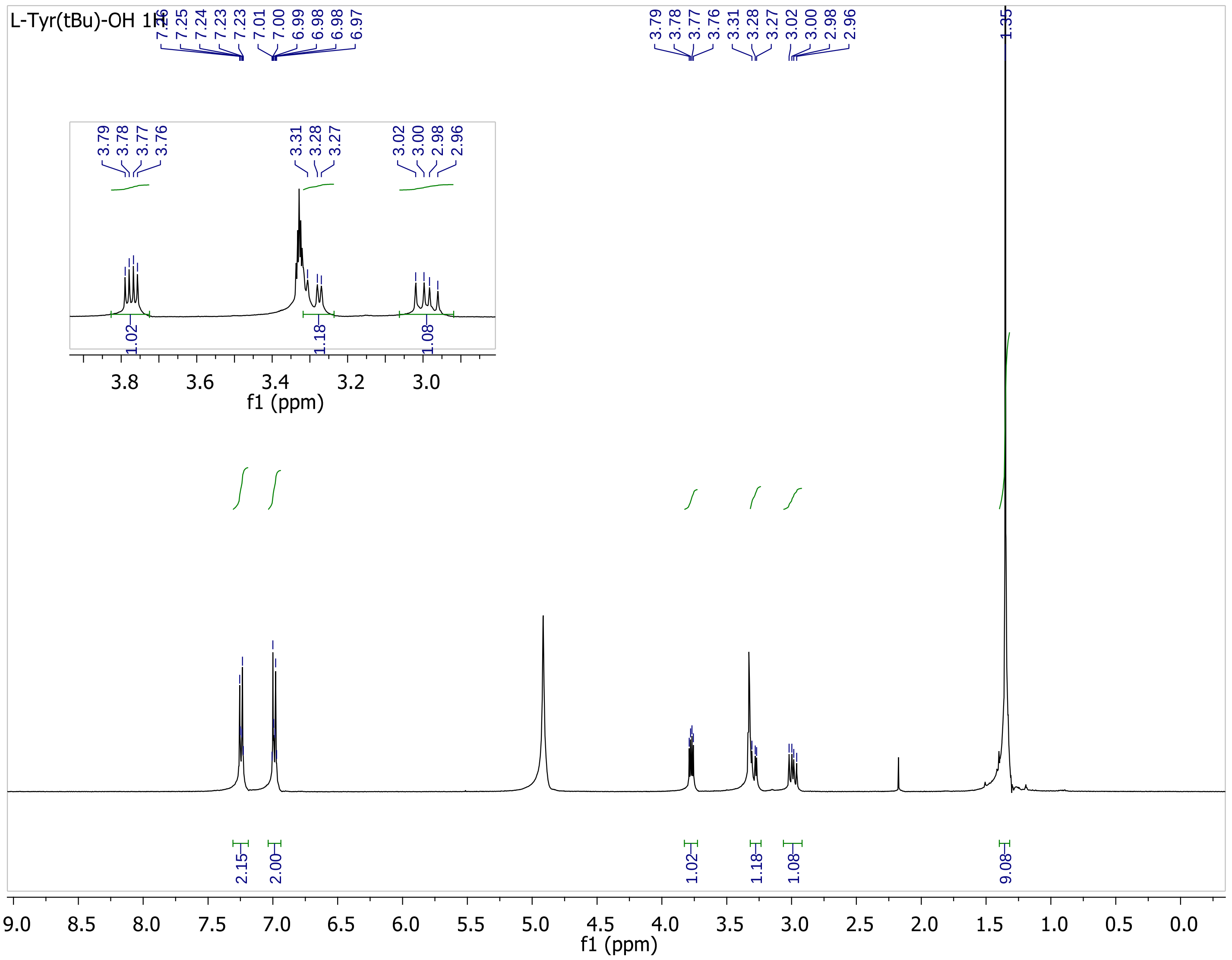

Figure S1. ^{1}H NMR spectrum of L-Tyr(*t*Bu)-OH (400 MHz, CD_3OD).

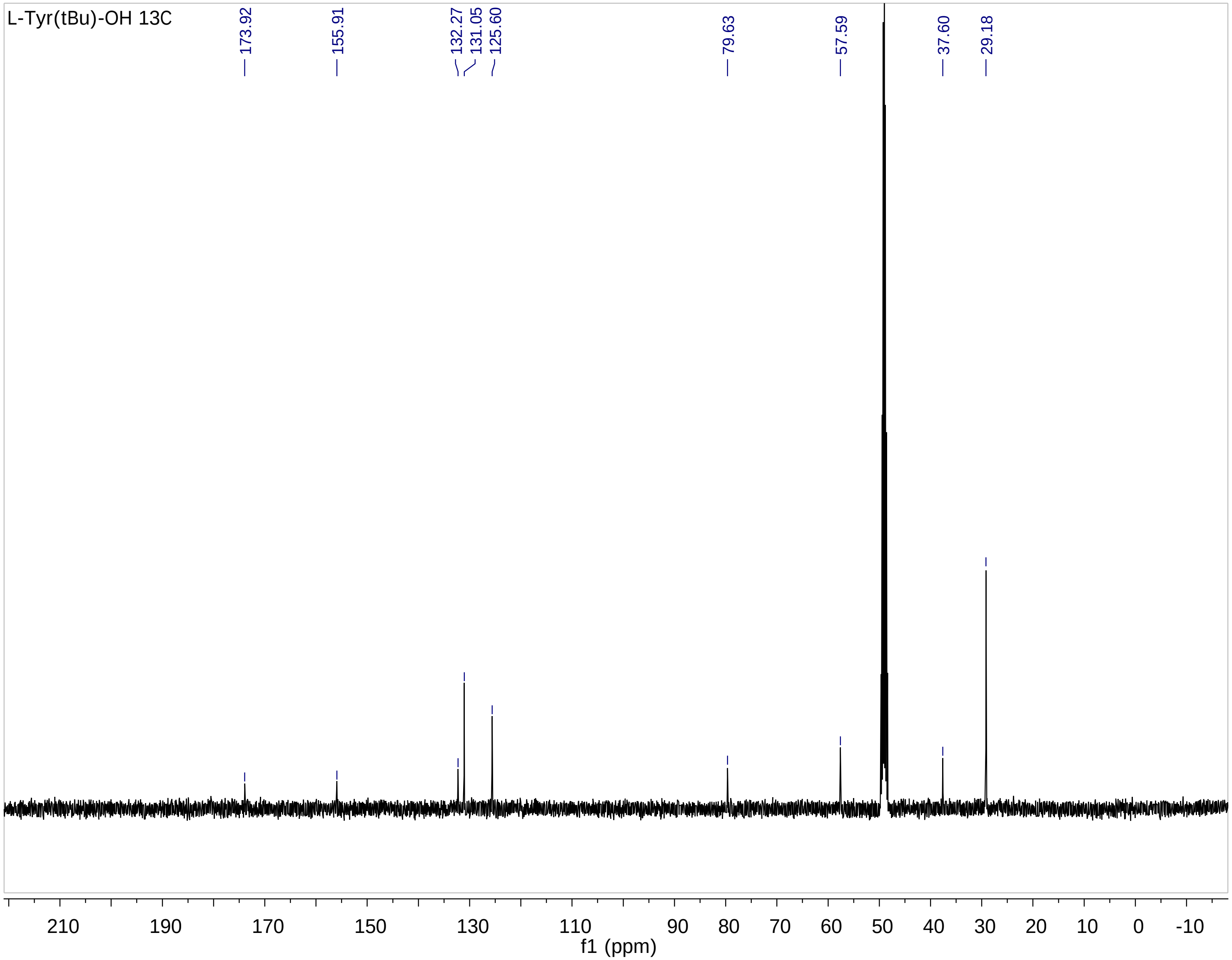

Figure S2. ^{13}C NMR spectrum of L-Tyr(*t*Bu)-OH (400 MHz, CD_3OD).

4. HPLC Analysis of L-Tyr(tBu)-OH

Purity of the L-Tyr(*t*Bu)-OH was analyzed by using Dionex Ultimate 3000 with Thermo Scientific Acclaim 120 reverse phase C18 column. The eluents are milliQ water with 0.1% TFA and ACN with 0.08% TFA. The method started from 1% ACN and increased to 90% ACN in 30 minutes.

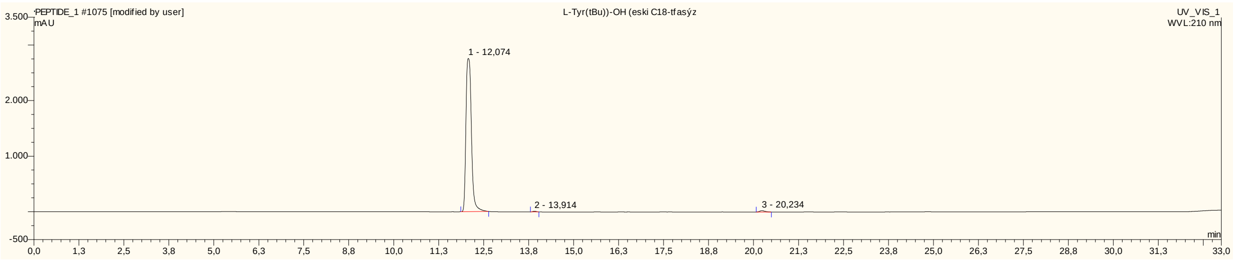

Figure S3. HPLC chromatogram of L-Tyr(*t*Bu)-OH (210 nm).

Table S1. Relative percentage of L-Tyr(*t*Bu)-OH from HPLC chromatogram. Retention time of L-Tyr(*t*Bu)-OH is 12.07 min.

No.	Ret.Time	Height	Area	Rel.Area
	min	mAU	mAU*min	%
1	12,07	2,757,099	488,526	98,91
2	13,91	11,286	1,372	0,28
3	20,23	22,998	4,027	0,82
Total:		2,791,382	493,926	100

5. ^{1}H and ^{13}C NMR Spectra of L-Tyr(TBDMS)-OH

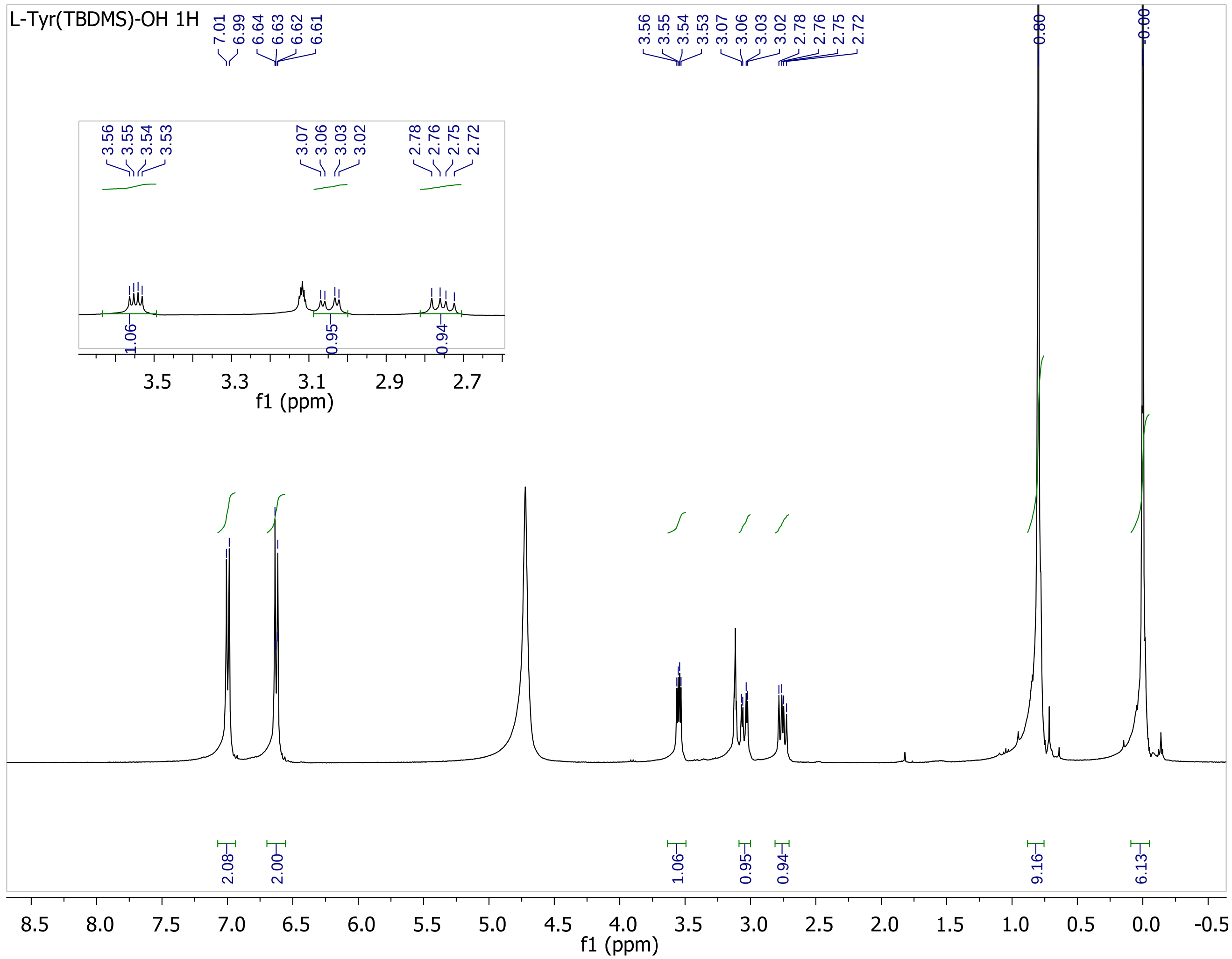

Figure S4. ^{1}H NMR spectrum of L-Tyr(TBDMS)-OH (400 MHz, CD_3OD).

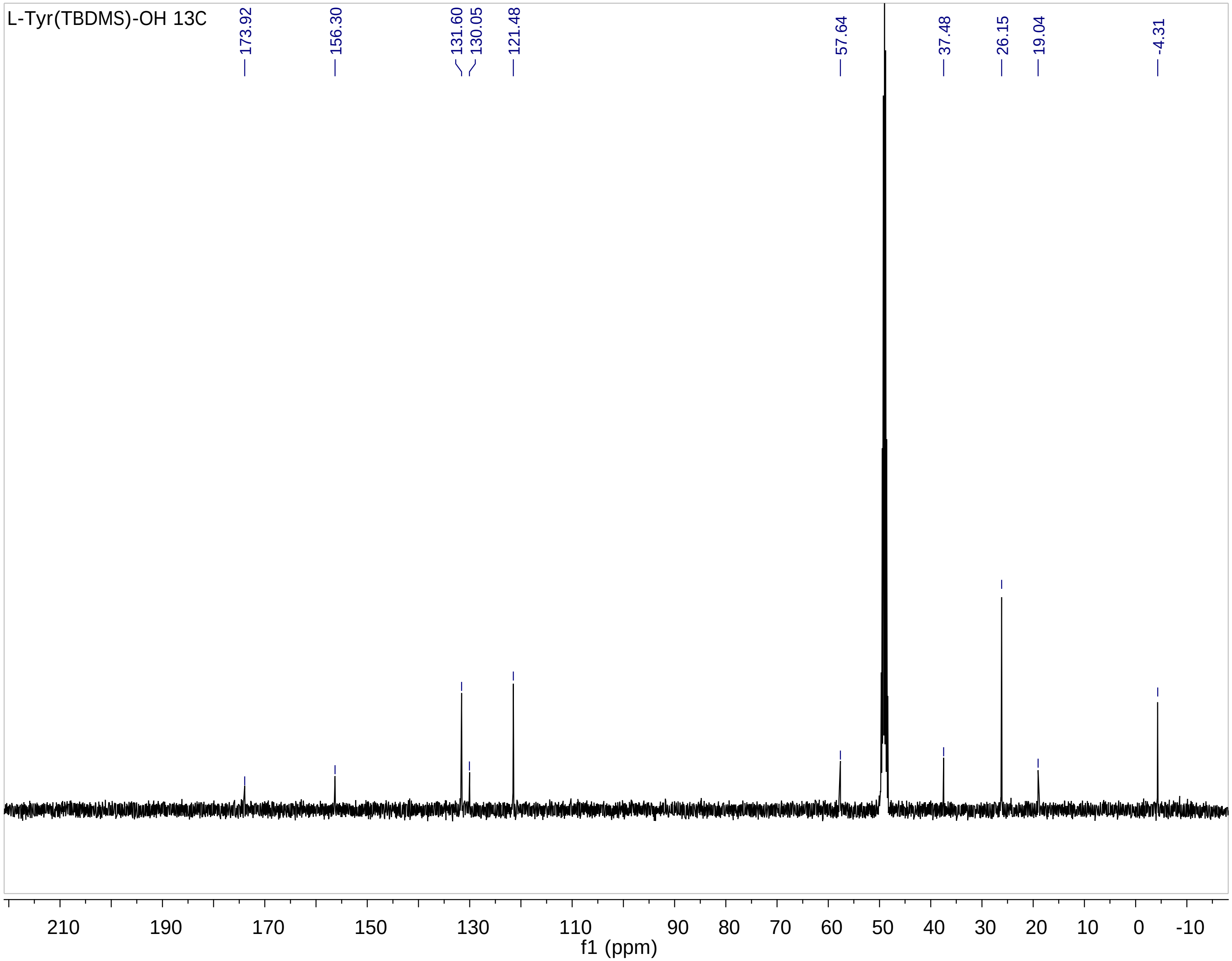

Figure S5. ^{13}C NMR spectrum of L-Tyr(TBDMS)-OH (400 MHz, CD_3OD).

6. *HPLC Analysis of L-Tyr(TBDMS)-OH*

Purity of the L-Tyr(TBDMS)-OH was analyzed by using Dionex Ultimate 3000 with Thermo Scientific Acclaim 120 reverse phase C18 column. The eluents are milliQ water with 0.1% TFA and ACN with 0.08% TFA. The method started from 1% ACN and increased to 90% ACN in 30 minutes.

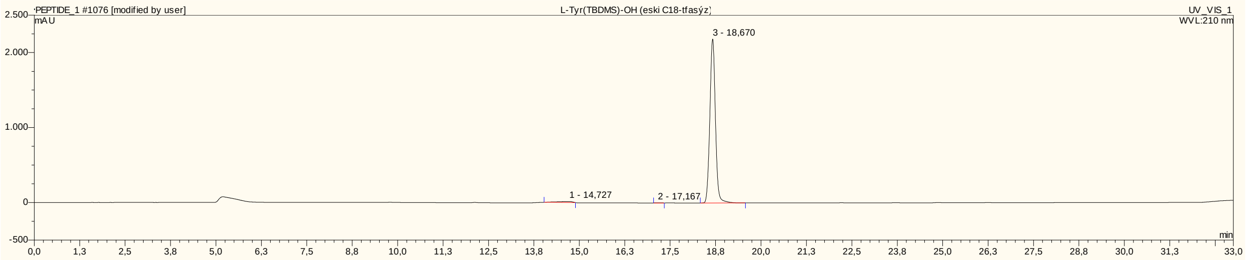

Figure S6. HPLC chromatogram of L-Tyr(*TBDMS*)-OH (210 nm).

Table S2. Relative percentage of L-Tyr(*t*Bu)-OH from HPLC chromatogram. Retention time of L-Tyr(*TBDMS*)-OH is 18.67.

No.	Ret.Time	Height	Area	Rel.Area
	min	mAU	mAU*min	%
1	14,73	13,214	6,656	1,69
2	17,17	1,232	0,183	0,05
3	18,67	2,187,603	386,698	98,26
Total:		2,202,049	393,537	100

7. *Modelling details and discussions*

All molecular dynamics simulations and analysis were performed using the Amber16/AmberTools17 program package.[3] Simulations in THF were performed for two systems: system 1 with only L-Tyr(*t*Bu)-OH molecules in their zwitterionic form (YTZ residues; net charge = 0) and system 2 containing an equimolar mixture of L-Tyr(*t*Bu)-OH molecules with deprotonated carboxylic group (YTB residues; net charge = -1) and protonated piperidine molecules (PPH residues; net charge = +1). GAFF parameters[4] were used for THF and PPH molecules, while the parameters for YTB and YTZ were adapted from the ff14SB force field.[5] The derivation of atomic point charges was performed for each newly defined residue following the RESP strategy[6] based on electrostatic potentials calculated at the HF/6-31G* level with Orca[7] on a Merz-Singh-Kollman grid,[8,9] accordingly with Amber force field standards. Parameters and library files are available as Supporting Information. Cubic simulation boxes with initial edges length of 56 Å were built with the packmol program.[10] System 1 contained 16 YTZ and 1300 THF molecules, while system 2 was made of 16 YTB, 16 PPH, and 1300 THF molecules.

After minimization of the simulation boxes, both systems were heated up and simulated by following the same protocol. For all steps of the molecular dynamics, the time step was 2fs, the SHAKE algorithm was applied to constrain hydrogen containing bonds, and particle mesh Ewald summation scheme was used with periodic boundary conditions and a nonbonded cut-off of 12 Å. The temperature was gradually increased, first in the NVT ensemble up to 100 K, and later in the NPT ensemble to reach the target temperature of 300 K. Temperature and pressure were controlled with Langevin dynamics (collision frequency of 4.0 ps^{-1}) and isotropic position scaling, respectively. The final density of both systems after heat up stabilized to 0.89 g cm^{-3}, consistently with the expected density of liquid THF. Production runs were then performed in the same NPT conditions for each system during 500 ns by saving coordinates every 20 ps. The first 250 ns of each simulations were disregarded from the subsequent analysis and considered as an equilibration period.

The simulations in THF were analyzed to identify stable assemblies using an in-house python script based on specific intermolecular distances. For system 1, two YTZ molecules were considered to belong to the same assembly if they featured a distance between their respective carboxylate carbon and ammonium nitrogen atoms shorter than 5 Å. This analysis was performed for each frame in the simulation to identify the largest and most stable assembly in terms of number of molecules and conservation of their identity. The frames and molecules corresponding to the identified assembly were further extracted and a structural clustering analysis based on the backbone atoms of each molecule only was performed to finally yield the representative structure depicted in Fig. S7a. This motif contained 9 YTZ molecules and was observed in all the frames (100%) within the last 250 ns of the simulation. In a similar manner, the

simulation of system 2 was analyzed based on the interatomic distance between carboxylic carbon atoms of YTB and the nitrogen atom of PPH molecules (as suggested by visual examination of the trajectories), with a cut-off of 5 Å to determine if two molecules belong to the same assembly. The representative structure of the largest and most stable assembly is depicted in Fig. S7b. It features 11 YTB and 11 PPH molecules and was observed for about 55 % of the last 250 ns in the simulation. A second, smaller but more stable assembly was observed for 93 % of the simulation and contained 5 YTB and 5 PPH molecules.

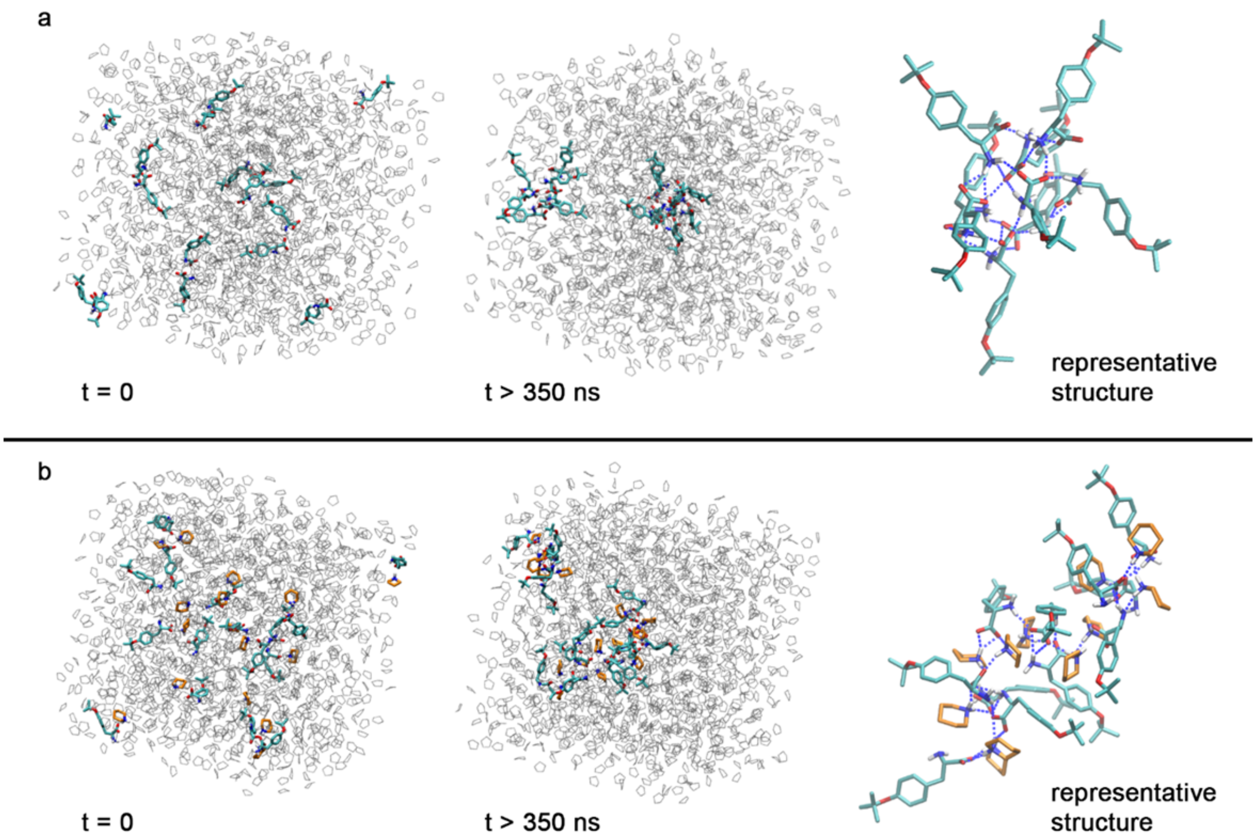

Figure S7. Representative configurations along the molecular dynamics simulation of L-Tyr(*t*Bu)-OH without (a) and with piperidine (b) in THF. In a and b, the left and middle panels respectively represent the simulation box at the beginning of the simulation and after a given period of time, showing the self-assembly of the solutes. The left panel depicts the representative structure obtain after identification of the largest, most stable assembly and clustering. THF molecules are represented with grey-coloured lines without hydrogen atoms. Carbon atoms of L-Tyr(*t*Bu)-OH and piperidine are coloured in cyan and orange, respectively. Only the hydrogen atoms of amine and ammonium groups are represented. Hydrogen bond interactions are shown with dashed blue lines.

Comparison of the assemblies obtained after simulation of systems 1 and 2 shows significant structural differences between the two models. In the case of system 1 (with L-Tyr(*t*Bu)-OH only) the molecules

interact through a dense network of hydrogen bonds between their carboxylate and ammonium groups. The corresponding assembly is highly stable, as it does not break once formed within the limit of the present simulation. In system 2, however, the assemblies are made of an alternation of L-Tyr(*t*Bu)-OH and piperidine molecules, where only the carboxylate part of the former is involved in the interactions that hold the molecules together. This results in a less dense network of hydrogen bonds, which in turn yields a less stable assembly. Most importantly, the motifs observed without and with piperidine are significantly different, involving interactions between different chemical groups. One would therefore expect such structures to yield significantly different XRD patterns. As the patterns of both samples, without and with piperidine, show the same signature, we conclude that piperidine does not have a structural role in the formation of the gel.

The representative structure of system 1 (without piperidine; figure S7a) was used to form an initial box of pure L-Tyr(*t*Bu)-OH. The initial motif of 9 YTZ molecules was replicated in all three Cartesian directions with translation vectors of 24, 24, and 18 Å of magnitude in the x, y, and z directions respectively, resulting in a simulation box containing a total of 243 YTZ molecules. Initial box parameters were set to 72, 72, and 54 Å to prevent steric clash. Then, minimization and heat up were performed using the same protocol as for the simulations in THF. The size of the simulation box spontaneously adapted during the NPT step to result in a dense packing of molecules without any vacuum hole. A production run was performed for 1 μs under the same conditions as for simulations in THF. Only the last 300 ns of the trajectory were analyzed to ensure that the simulation reached equilibrium.

Analysis of the connectivity between molecules in pure L-Tyr(*t*Bu)-OH resulted in the identification of three long networks containing 135, 81, and 27 YTZ molecules, which were found to be stable for 93-100 % of the analysis time-window. As discussed in the main text, the molecules assemble in a fibre-like fashion with a core consisting in a dense and tight network of hydrogen bonds, and an outer part made of the hydrophobic tails of the gelator. The fibres mainly interact through hydrophobic interactions between the *tert*-butyl groups, with some occurrences of hydrogen bond bridges.

Figure S9 shows the radial distribution function (RDF), g(r), for selected pairs of atoms. The distributions were normalized using a density of 0.0028228 molecules Å-3, as derived from the average density of the simulation box within the last 300 ns (1.1113 g cm^{-3}). Only intermolecular distances were considered in the distributions. RDF representing π–π interactions were calculated based on the distance between the centre of mass of each aromatic ring. The inset in Figure S8 indicates the nomenclature of atoms used to label the different plots. The RDF labelled as "*t*Bu" corresponds to the distances between

each pair of methyl carbon atoms in the simulation box (C2, C3, and C4). Similarly, the O-O distribution was calculated including all pairs of carboxylate oxygen atoms (O1 and O2).

With the exception of CB-CB and π–π RDFs, all distributions feature well-defined and intense peaks, showing a clear and periodic arrangement of the molecules in the simulation box. The absence of structuration for CB-CB and π–π distributions indicates that the interaction of side chains is mainly dictated by the hydrophobic contact between *tert*-butyl groups and that no π–π interaction occurs. The tail of the side chains give rise to a well-organized packing as depicted by the fairly intense peak centered at about 6 Å in the C1-C1 RDF. The pairs of atoms in the hydrophilic part of the molecule show a tight and well-structured packing. In particular, C-C and N-N distributions feature highly structured peaks, suggesting the repetitive occurrence of several sets of interatomic distances. This last observation is consistent with the high concentration of diffraction peaks in the XRD pattern at angles corresponding to distances shorter than 7 Å. The most intense and sharp peak is observed for the C-N RDF, reflecting the tight interaction between carboxylate and ammonium groups of the molecules. The first peak in this distribution is centered around 3.33 Å, which is compatible with the most intense diffraction on the XRD pattern measured at 2θ = 26.65° (d_{hkl} = 3.34 Å).

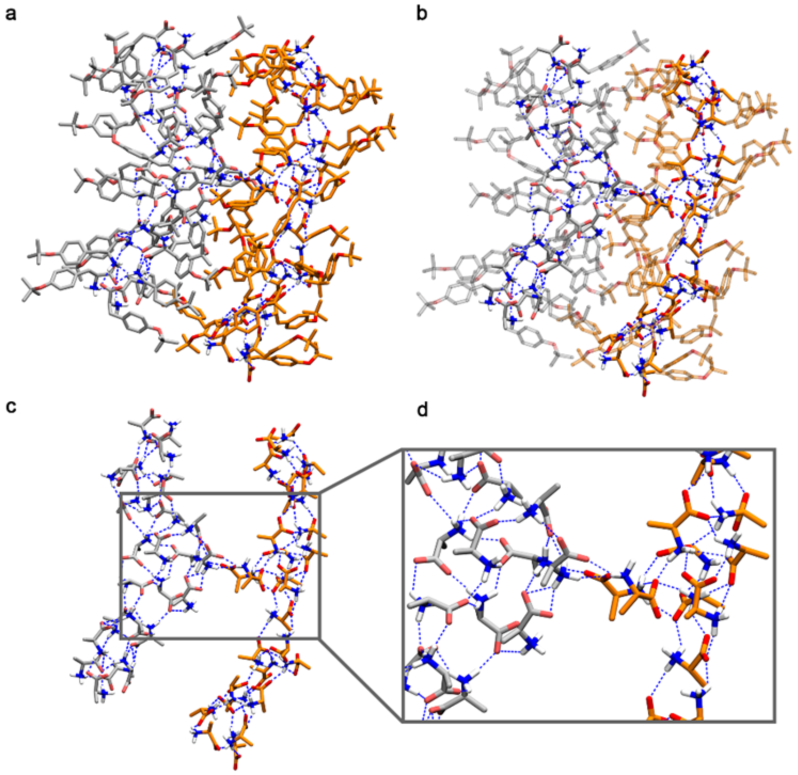

Figure S8. Alternative representation of the fibre-like structure depicted in Fig. 4c of the main text, as obtained after one microsecond MD simulation of pure L-Tyr(*t*Bu)-OH. The molecules were arbitrarily

assigned to a given fibre and accordingly coloured in grey and orange colours. The four panels show various representation with all atoms except side chain hydrogens in panels a and b, and only backbone atoms in panels c and d. Side chain atoms are shown in transparent in panel b to highlight the network of hydrogen bonds. Panel d is a magnification of the branching region between the two fibres.

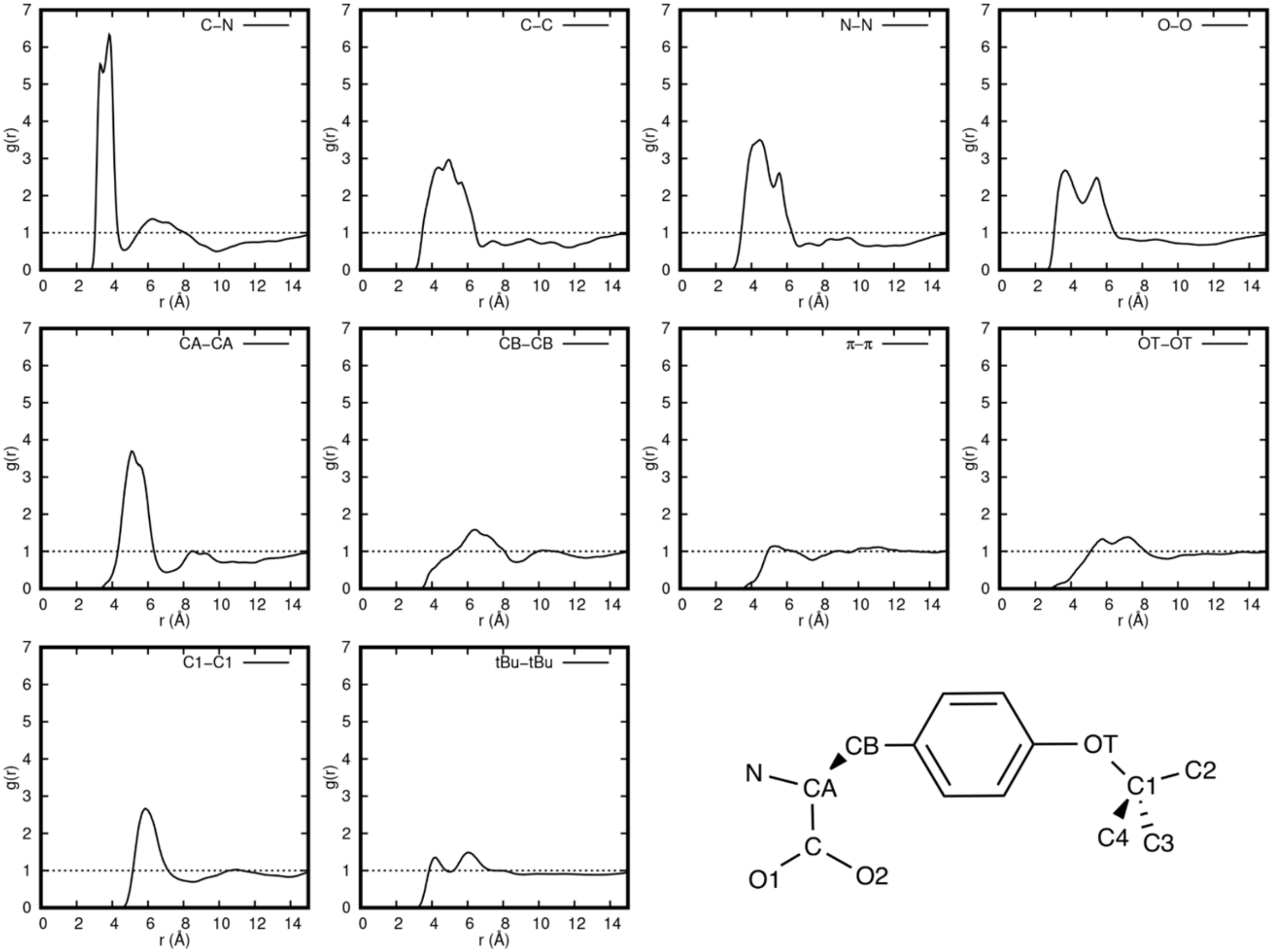

Figure S9. Radial distribution functions (RDFs) for selected pairs of atoms, as calculated from the last 300 ns of the 1 µs-long simulation of pure L-Tyr(tBu)-OH. The inset at the bottom-right depicts the nomenclature used to label non-hydrogen atoms.

References

1. T. Albert, T. Richmond, M. Rodesch, K. P. Stengele, J. Buehler, M. Ott *U.S. Pat.*, 9 346 892, 2016.
2. D. A. Case, D. S. Cerutti, T. E. Cheatham, III, T. A. Darden, R.E. Duke, T. J. Giese, H. Gohlke, A. W. Goetz, D. Greene, N. Homeyer, S. Izadi, A. Kovalenko, T. S. Lee, S. LeGrand, P. Li, C. Lin, J. Liu, T. Luchko, R. Luo, D. Mermelstein, K. M. Merz, G. Monard, H. Nguyen, I. Omelyan, A. Onufriev, F. Pan, R. Qi, D. R. Roe, A. Roitberg, C. Sagui, C. L. Simmerling, W. M. Botello-Smith, J. Swails, R. C. Walker, J. Wang, R. M. Wolf, X. Wu, L. Xiao, D. M. York and P. A. Kollman (2017), AMBER 2017, University of California, San Francisco.
3. J. Wang, R. M. Wolf, J. W. Caldwell, P. A. Kollman, D. A. Case *J. Comput. Chem.*, 2004, **25**, 1157.
4. J. A. Maier, C. Martinez, K. Kasavajhala, L. Wickstrom, K. E. Hauser, C. Simmerling *J. Chem. Theory Comput.*, 2015, **11**, 3696
5. C. I. Bayly, P. Cieplak, W. D. Cornell, P. A. Kollman, *J. Phys. Chem.* 1993, **97**, 10269.
6. F. Neese, *WIREs Comput. Mol. Sci.*, 2012, **2**, 73.
7. U. C. Singh and P. A. Kollman, *J. Comput. Chem.*, 1984, **5**, 129.
8. B. H. Besler, K. M. Merz Jr., P. A. Kollman, *J. Comput. Chem.*, 1990, **11**, 431.
9. L. Martínez, R. Andrade, E. G. Birgin, J. M. Martínez., *J. Comput. Chem.*, 2009, **30**, 2157.
